# Labial Frenectomy Using Conventional Scalpel Technique: A Case Report

**DOI:** 10.7759/cureus.64436

**Published:** 2024-07-12

**Authors:** Akansha A Rai, Shrishti S Salian, Prasad v Dhadse, Ruchita T Patil, Sanehi D Punse, Kaveri M Paleriya

**Affiliations:** 1 Department of Periodontics and Implantology, Datta Meghe Institute of Higher Education and Research, Wardha, IND

**Keywords:** scalpel, orthodontic treatment, midline diastema, abnormal frenum, conventional frenectomy, frenectomy

## Abstract

Frenum morphology is of pivotal concern while treating patients who desire improved aesthetics and for treating patients with midline diastemas, as it may lead to failure or relapse of orthodontic treatment. Frenum, a thick band of muscle fiber, may present with abnormalities in the shape, size, form, number, and thickness, which may cause gingival recession along with poor oral health. Frenectomy is adopted as the plan of action while treating abnormal frenum cases by resecting the frenum attachment to provide closure of the spacing between the upper anterior teeth, as in the case of midline diastema. Various forms and techniques of frenectomy have been adopted according to the type of frenum attachment and aesthetic concerns of the patients. Amidst the various treatment options available, conventional frenectomy using a scalpel has emerged as a viable solution for treating patients. The surgeons value and praise its exceptional precision and ease of use, and the patients prefer it for its affordability; hence, a functional and aesthetic outcome is achieved via this treatment. This report provides a comprehensive overview of a case of conventional frenectomy with a one-week follow-up.

## Introduction

Frenum, a thin band of tissue usually noticed in the anterior region of the jaw used to connect the upper lip, consists of two types: the lingual frenum and the labial frenum [1]. The abnormal frenum attachment can lead to poor oral hygiene as it provides obstruction while toothbrushing and further muscle pull. Rigorous attempts to clean and brush can also cause problems like gingival recession [2]. It can lead to aesthetic problems like midline diastema, which causes spacing between the upper anterior teeth, resulting in poor aesthetics and compromising the orthodontic treatment of the patient, further leading to a relapse of the treatment. In patients with complaints of sustaining midline diastema, the high and thick labial frenum must be excised. Thus, it is indicated in cases where there is a presence of high frenum attachment leading to midline diastema, in cases of gingival recession, and where shallow vestibule provides a hindrance in maintaining good oral hygiene [3]. The frenum attachments have been classified into four major types, which consist of mucosal, gingival, papillary, and papilla-penetrating, by Mirko et al. in 1974 [4]. In the mucosal type, the frenal fibers are attached to the mucogingival junction. In the gingival type, the fibers are inserted within the attached gingiva. Papillary is when the fibers extend into the interdental papilla, and papilla-penetrating is when the frenal fibers cross the alveolar process and extend up to the palatine papilla. The diagnosis of the type of frenum is made by applying tension to the frenum attachment. In a tension test, one can thoroughly visualize and diagnose abnormal frenum by examining the papillary tip or by observing the presence of blanching induced by regional ischemia along with changes in the color and texture of the interdental papilla. These features are a positive finding for abnormal frenum attachment and, therefore, give a positive tension test result [5]. The abnormal frenum can be corrected with procedures known as frenectomy or frenotomy. The term frenotomy, which is also commonly used, includes creating an incision to move the frenum attachment, unlike frenectomy, which consists of a complete excision of the resected frenum. Over time and with the advancement of technology, various modalities have been developed to effectuate the process of frenectomy. It can be performed by conventional methods using a blade and scalpel, soft tissue laser, or electrocautery. The conventional frenectomy techniques employed include conventional (classical) frenectomy, Miller's technique, V-Y plasty, Z-plasty, and frenectomy by electrosurgery and laser [6]. In this case, the patient underwent a standard scalpel frenectomy.

## Case presentation

**Figure 1:**
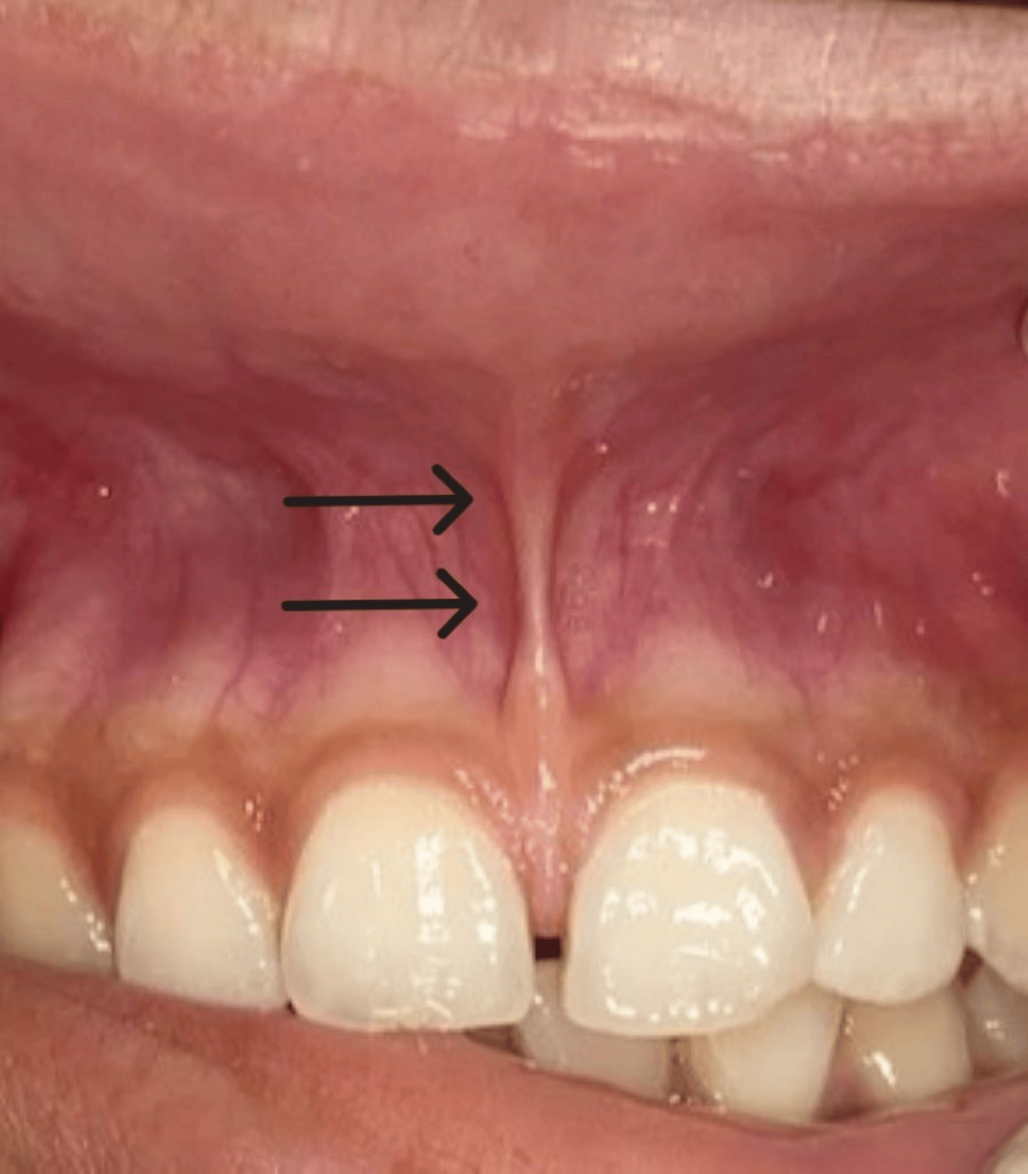
Pre-operative photograph showing papilla-penetrating type of frenum attachment.

A 22-year-old female patient came to the Department of Periodontics at Sharad Pawar Dental College and Hospital (SPDC&H) with the chief complaint of spacing between upper anterior teeth causing disparaged aesthetics. On clinical examination, a tension test was performed, in which tension was applied over the area to see the movement of the papillary tip or blanching produced due to ischemia of the region which was detected visually. The test revealed a papilla-penetrating type of frenum attachment between tooth numbers 11 and 21 (Figure 1). The medical history was not significant. The patient was not on any immunosuppressive, anti-hypertensive, anti-asthmatic, or anti-diabetic drugs. The patient had no significant dental history, and on intra-oral examination, no abnormalities were detected. The gingival level was adequate, and no enlargement or inflammation of the gingival region was seen. The patient was briefed about the surgical procedure, and after obtaining written consent from the patient, a labial frenectomy procedure was planned using a classical technique involving the use of a scalpel.

After undergoing an ultrasonic scaling procedure, the patient was referred to the Department of Oral Pathology for blood investigations. The reports of hemoglobin (Hb) levels, bleeding time (BT), and clotting time (CT) were normal (Table 1). After confirming the hematological findings of the patient, the labial frenectomy procedure was carried forward. 

**Table 1:** Hematological reports of a 22-year-old female patient. Hb: hemoglobin; BT: bleeding time; CT: clotting time

Hematological data	Patient values	Normal values (female)	Normal values (male)
Hb%	13 gm%	11-14.5 gm%	12-15.5 gm%
BT	2 minutes 22 seconds	1-3 minutes	1-3 minutes
CT	3 minutes 51 seconds	1-5 minutes	1-5 minutes

**Figure 2:**
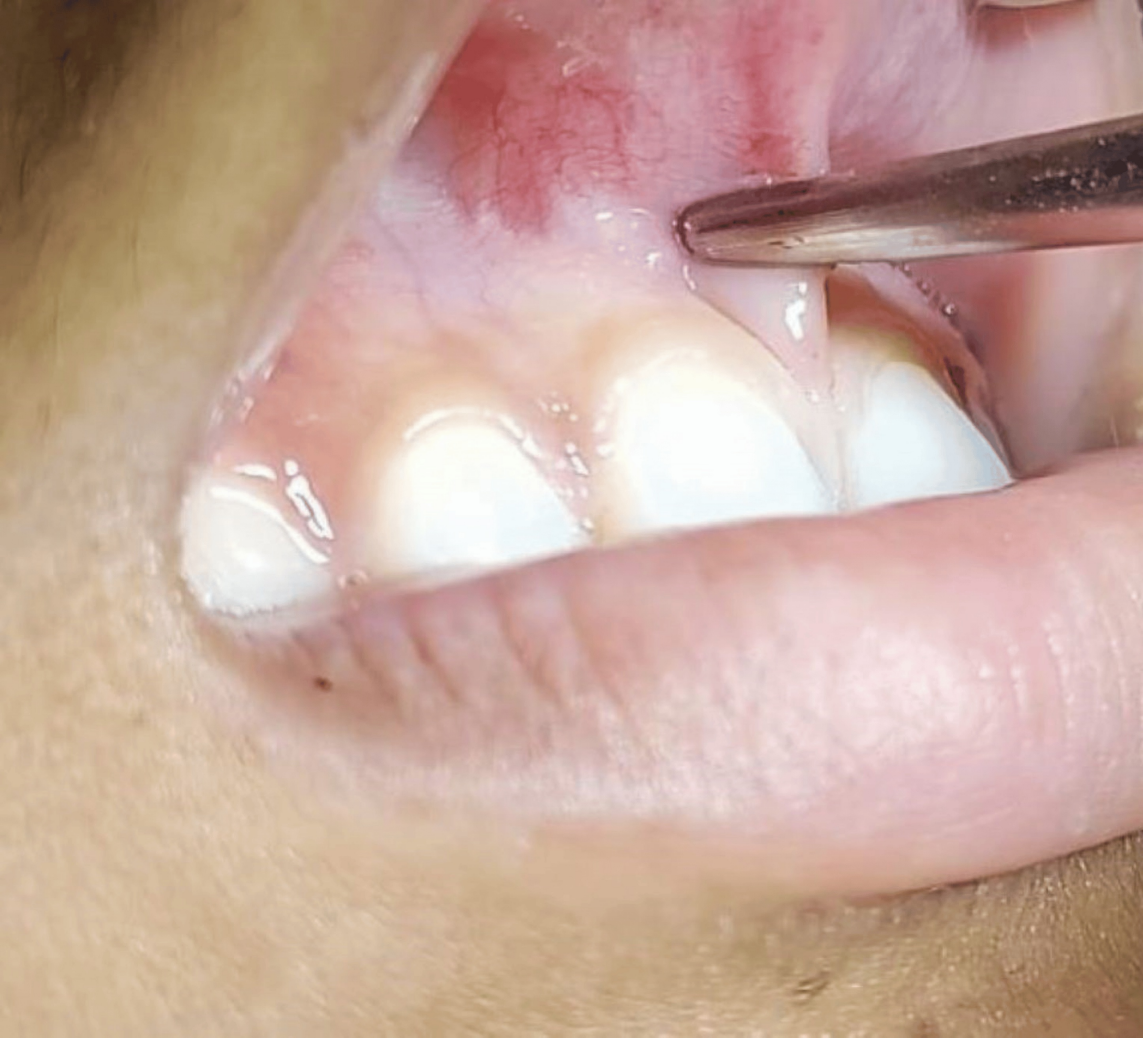
Frenum held with a hemostat.

Local infiltration was given using 2% lignocaine combined with 1:80,000 adrenaline (LOX 2% Adrenaline) and was used to anaesthetize the frenum and surrounding tissues. The frenum was held with a hemostat until the furthest depth of the vestibule (Figure 2).

**Figure 3:**
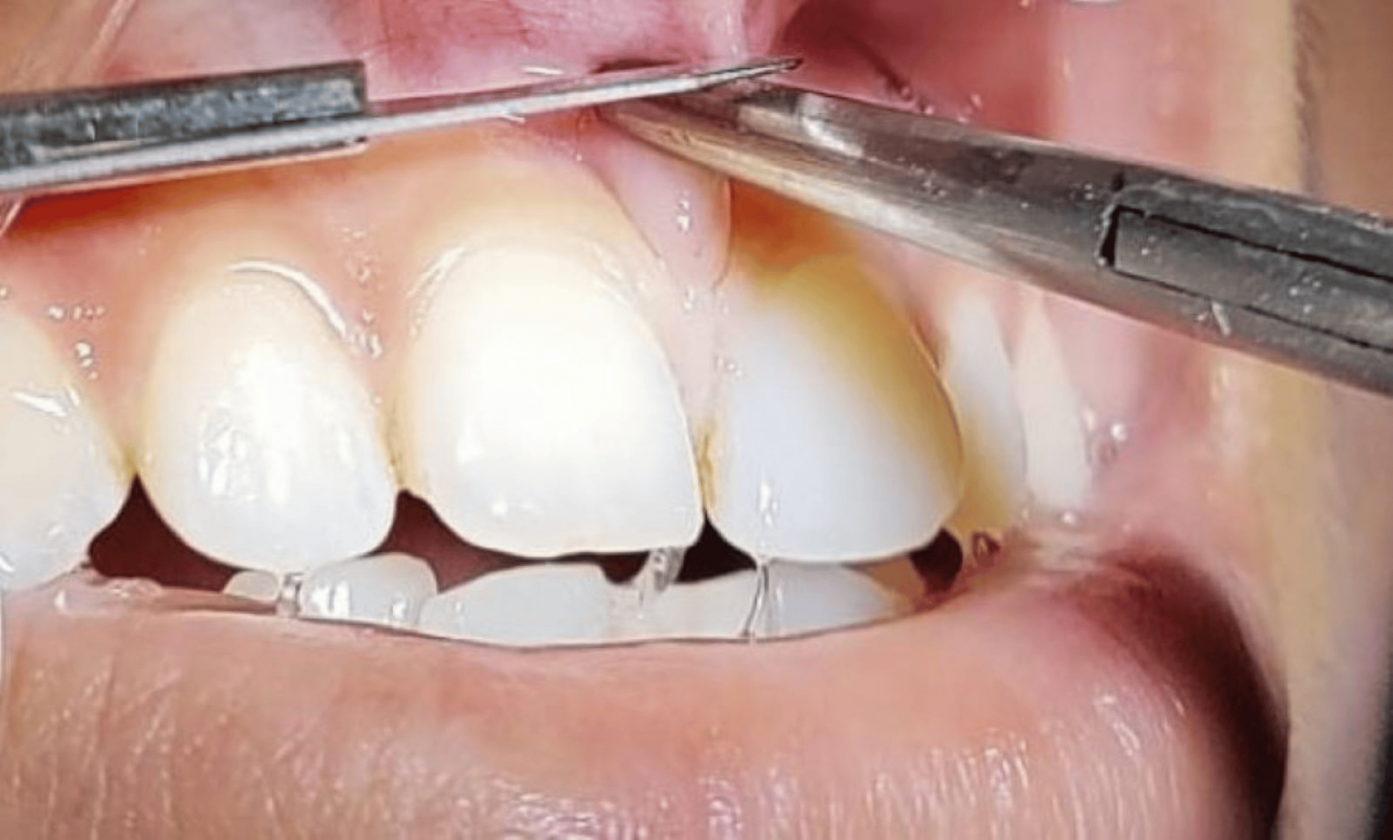
A wedge-shaped incision was given.

Using a No. 15 Bard-Parker blade, a wedge-shaped incision was made using a scalpel. The entire frenum was resected, and the underlying tissue was exposed (Figure 3).

**Figure 4:**
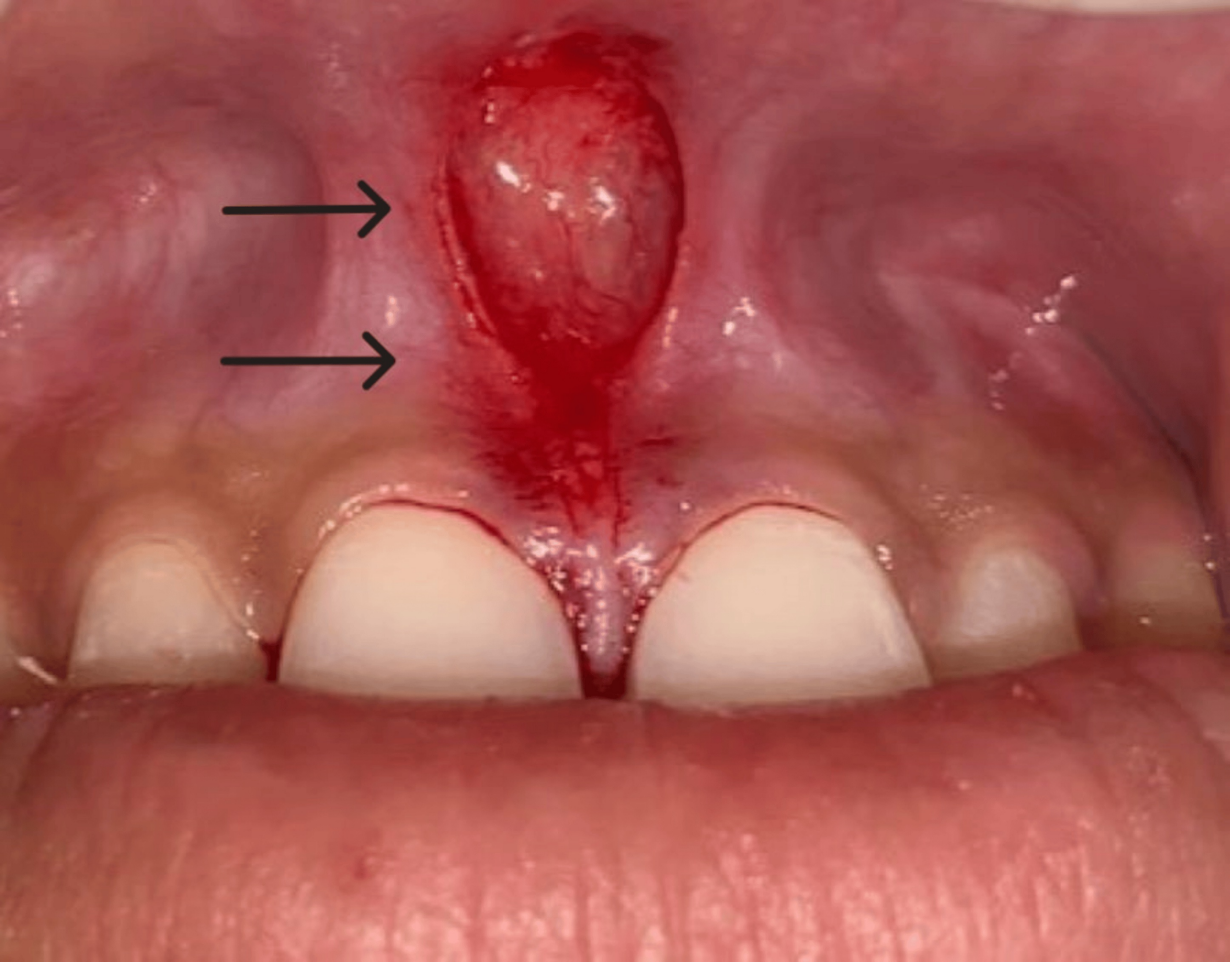
Frenum along with its alveolar attachment was removed, and a pear-shaped surgical area was seen.

The whole thick band of fibrous tissue, including its alveolar attachment, was excised, and a pear-shaped surgical area was seen. The surgical site was then irrigated with normal saline, and under all aseptic conditions, adequate hemostasis was achieved (Figure 4). 

**Figure 5:**
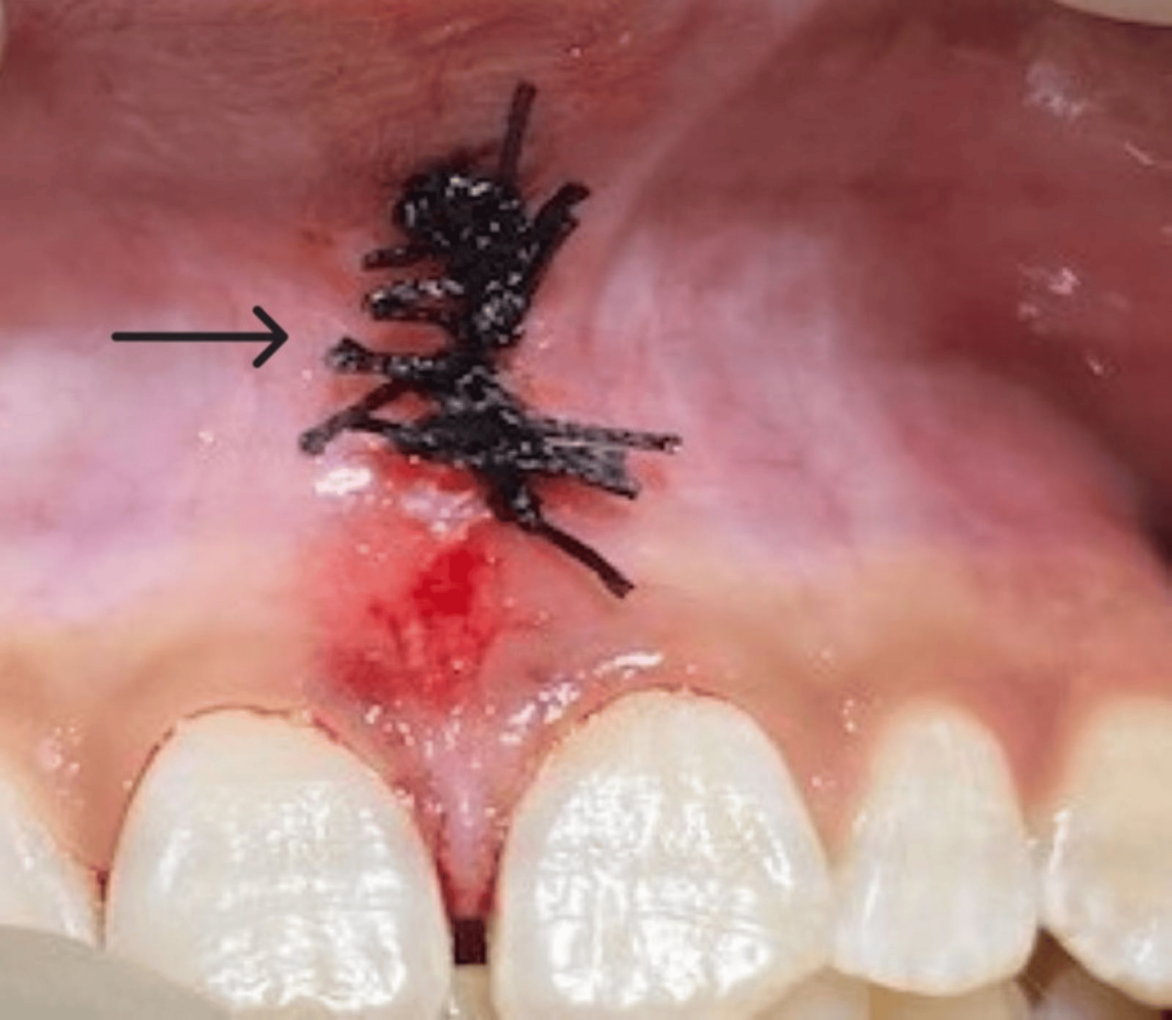
Simple interrupted sutures were given.

3-0 silk sutures were used, and simple interrupted sutures were given (Figure 5). COE-PAK was placed to provide the necessary periodontal dressing over the region. The patient was provided with post-operative oral hygiene instructions and was prescribed medications (Aceclofenac + paracetamol 500mg, SOS) for the same. After seven days, the patient was recalled back to the department for further re-evaluation. 

**Figure 6:**
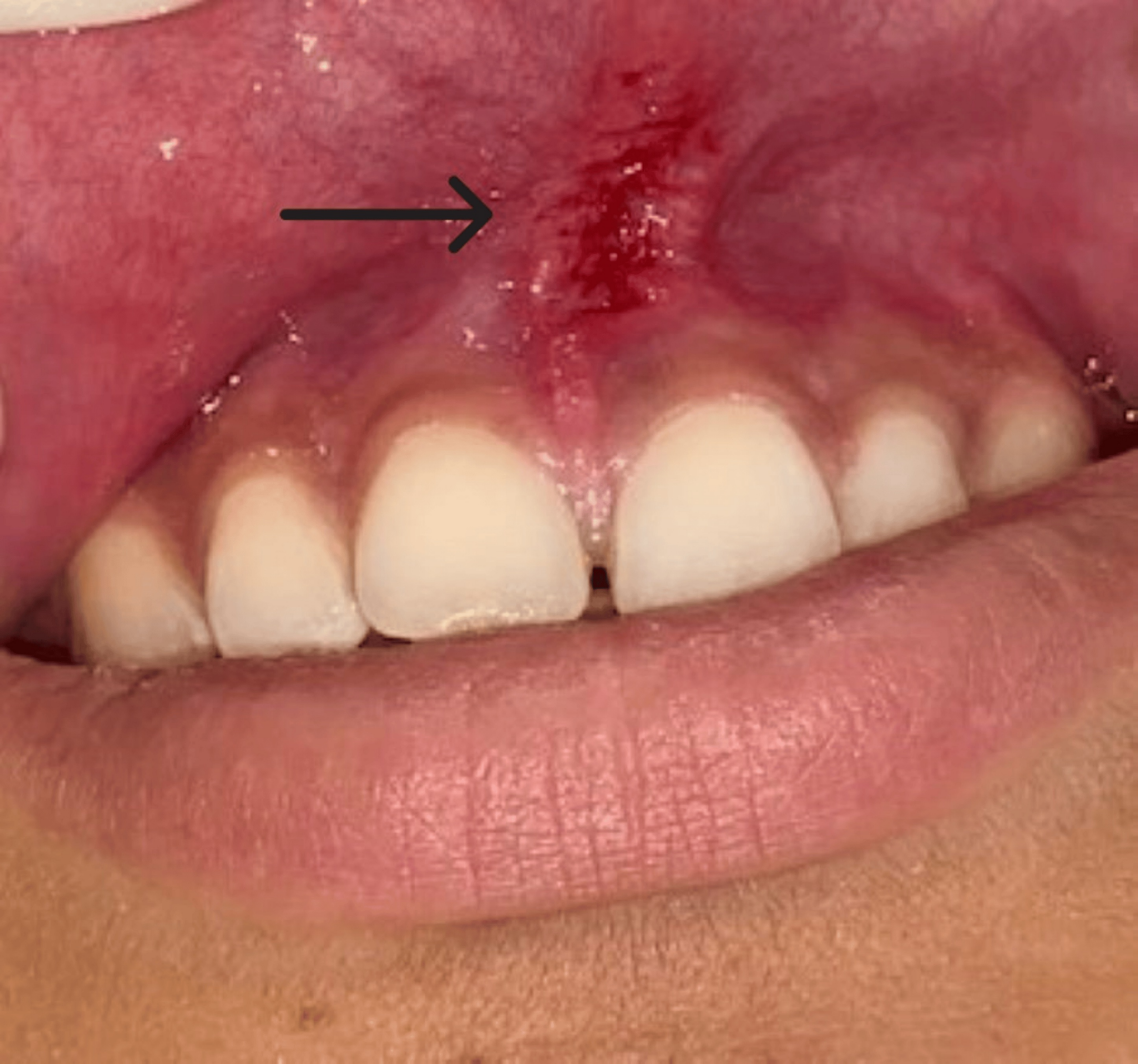
Post-operative photograph seen on the seventh day.

On the seventh day, the patient was recalled back to the Department of Periodontics for suture removal. After seven days, the patient showed satisfactory results. Significant healing of the area was seen, and the patient did not complain of any pain, and no presence of edema was seen. The patient presented with improved aesthetics, and there were no significant disturbances in speech or mastication from the anterior region of the jaw. Hence, a favourable outcome was achieved, and the patient was satisfied with the treatment (Figure 6).

## Discussion

Midline diastema can lead to poor appearance and provide a hindrance in orthodontic treatment. The presence of a wide and thick frenum attachment, which is a persistent cause of spacing between the upper anterior teeth, has to be treated as it may lead to failure or relapse of the orthodontic treatment. This can be corrected by frenectomy or frenotomy techniques [7]. Archer and Kruger introduced the traditional approach between 1961 and 1964 [8]. This surgery is utilised to ensure the removal of the muscle fibers presumed to be joining the palatine papilla and orbicularis oris. The spacing present can be seen in the deciduous dentition or primary dentition. In deciduous dentition, this spacing is in the form of either physiological spacing or pathological spacing. In the primary dentition phase, children may have generalised spacing, localised spacing, no spacing, or a crowded dentition. Numerous causes pertaining to midline diastema include improperly developing dentition, an imperfect fusion of the midline of the premaxilla, tooth material deficiency as in the case of microdontia, micrognathia, peg laterals, mesiodens, and physical impediments such as retained deciduous teeth and abnormal labial frenum. Pathological migration of the tooth, odontomas occurring in the midline, and developmental cysts in the oro-facial midline are also some of the pathological causes of midline diastema. Some habits also contribute to midline diastema. These habits include thumb sucking, tongue thrusting, and frenum thrusting. Multiple techniques have been developed to provide speedy recovery to the patient and also to advocate early healing of the diamond-shaped or pear-shaped scar. Methods such as Z-plasty and lasers are used to provide an easy and efficient outcome. However, the choice of method used for frenectomy depends on the functional and aesthetic outcome desired by the patient and also on the type of frenum attachment. However, the majority of operations use surgical frenectomy approaches such as the traditional (classical) technique and do not take into account appearances or the extent of the associated gingiva. The scalpel technique was originally preferred as it provides certain advantages such as ease of use, precision, affordability, and better wound healing. Surgeons praise its exceptional control and aptitude to retain its structural integrity throughout procedures. It may be easier to perform, but there is a downside to the conventional frenectomy technique, which is that it leaves a longitudinal incision and causes scar formation, which may be unaesthetic to the patient. The conventional method heals with primary intention, resulting in little scar development. Initial bleeding occurs, followed by epithelial cell migration and proliferation, which cause cut wound ends to approximate. There is also no granulation tissue formation [9]. Hence, this scar formation contradicts conventional frenectomy prior to orthodontic treatment. A few other drawbacks of employing a scalpel include the necessity for suturing, insufficient hemostasis, and unfavourable consequences following surgery such as pain, edema, and uneasiness [10]. However, even though methods such as Z-plasty and lasers provide little scar formation with satisfactory and desired healing, the scalpel technique will always continue to be the method of choice for treating orthodontic patients due to its precision, ease of use, and affordability [11].

## Conclusions

An eminent periodontal surgery, frenectomy, is of crucial importance in cases that need orthodontic correction. It has been a noteworthy procedure for providing for and fulfilling the aesthetic requirements of the patient. The periodontist proves to be of service and joins forces with an orthodontist to provide a satisfactory outcome for the patient who desires an endearing smile. As seen in this case, the conventional frenectomy procedure was used. Many may resort to using lasers and other modern modalities of performing frenectomy procedures; however, the conventional method still stands strong in providing precision and ease of operation to the surgeon, as well as proving to be affordable for the patient. The above case shows satisfactory healing on recall within seven days and aids in the success of the orthodontic treatment. The positive result achieved in this case proves that, over time, the conventional blade and scalpel method continues to prove its usefulness and reliability in treating high-frequency cases. Its post-operative outcome proved to be painless for the patient and yielded optimal function, durability, and aesthetic outcome. This case validates that, over time, conventional frenectomy techniques have continued to remain the standard choice of surgeons and will continue to yield promising outcomes. 
